# Using a biologically annotated library to analyze the anticancer mechanism of serine palmitoyl transferase (SPT) inhibitors

**DOI:** 10.1002/2211-5463.12196

**Published:** 2017-02-13

**Authors:** Osamu Sano, Ken‐ichi Kazetani, Ryutaro Adachi, Osamu Kurasawa, Tomohiro Kawamoto, Hidehisa Iwata

**Affiliations:** ^1^ BioMolecular Research Laboratories, ResearchTakeda Pharmaceutical Company Ltd.FujisawaJapan; ^2^ Oncology Drug Discovery Unit, ResearchTakeda Pharmaceutical Company Ltd.FujisawaJapan

**Keywords:** biologically annotated library, combination screening, COX‐2, necrosis, serine palmitoyl transferase

## Abstract

Mechanistic understanding is crucial to anticancer drug discovery. Here, we reveal that inhibition of serine palmitoyl transferase (SPT), the rate‐limiting enzyme in sphingolipid synthesis, induced death in a lung cancer cell line via a necrosis‐dependent pathway. To elucidate the mechanism of cell death induced by SPT inhibition, a biologically annotated library of diverse compounds was screened with an SPT inhibitor. This analysis identified suppressors of SPT inhibitor‐mediated cell death. Further analysis using hit compounds from this screening revealed that SPT inhibitors induce COX‐2 expression, leading to necrosis‐dependent cell death. SPT inhibitors might therefore represent novel candidates for cancer therapy via necrosis pathway regulation. Our data illustrate that compound combination screening of biologically annotated libraries could be used for mechanistic elucidation.

**Abbreviations**
COX‐2: cycloxygenase‐2
LDH: lactate dehydrogenase
MOA: mechanisms of action
SPT: Serine palmitoyl transferase

Cancer is a major public health program worldwide, and accordingly, pharmaceutical companies aim to develop novel anticancer‐related drugs. Although both genetic and environmental factors are closely related to cancer development and are responsible for some portion of cancer progression, many as yet undescribed factors are also involved in cancer progression. In addition to conventional medical and radiation treatments, molecular targeted therapy has recently become popular in the drug discovery process. Especially, developing anticancer drugs regulating metabolic pathways that are selectively activated in cancer cells represent a new promising approach to cancer therapy.

Cancer metabolism is the focus of current and emerging therapeutic approaches to anticancer drug discovery 1, 2, 3. The best known example of a metabolic shift in cancer cells is the Warburg effect 4. Cancer cells tend to depend on the glycolytic pathway rather than the tricarboxylic acid (TCA) cycle in order to generate energy more efficiently in a hypoxic microenvironment. Recent metabolomics technology research has revealed additional metabolic pathways that are closely related to cancer cell growth. Newly identified cancer metabolism‐related targets, such as isocitrate dehydrogenase 1 (IDH1) and HMG‐CoA reductase, are now considered promising anticancer drug targets 5, 6.

Serine palmitoyl transferase (SPT) mediates the conjugation of serine and palmitoyl CoA to form ceramide and represents a rate‐limiting step in sphingolipid synthesis. Ceramide is a well‐known cytotoxic lipid that under normal conditions, is readily transferred from the endoplasmic reticulum (ER) to the Golgi by the ceramide transfer protein CERT, where it undergoes further synthesis to glucosylceramide, sphingomyelin, and sphingosine‐1‐phosphate 7, 8. Abnormal sphingolipid metabolism has been observed in several types of cancer cells. In head and neck cancer, ceramidase overexpression enhances resistance to Fas ligand‐mediated apoptosis, and in various solid cancers, sphingosine kinase 1 overexpression leads to enhanced proliferation 9, 10, 11.

Although the metabolomics approach to cancer drug discovery works well and has led to the identification of new anticancer drug targets, the relationship between metabolic alterations and cancer cell growth is not always clear, and it is therefore critical for drug discovery researchers to understand the mechanisms of action (MOA) for such drugs. There are two commonly accepted methods for analyzing the MOA of cancer drugs. One method involves a target‐specific, hypothesis‐based approach that combines known information with newly obtained data from transcriptome and metabolome analyses. The other method involves a discovery‐based approach involving a functional genomics analysis of whole‐genome siRNA or shRNA 12, 13. Functional genomics analyses have provided many novel possibilities with regard to target relationships and thus represent a powerful approach for the identification of relatively novel targets. However, these unbiased siRNA‐ or shRNA‐based approaches share the fundamental challenges of off‐target effect 14, 15, knockdown efficiency, protein turnover, and compensatory reactions 16. The establishment of alternative methods would be valuable to our understanding of the MOA of anticancer drugs.

Biologically annotated library screening is currently attracting considerable interest as a straightforward approach to phenotypic drug discovery 17, 18, 19, 20, 21, 22. This approach allows us to easily link target molecules with disease phenotypes and to generate hypotheses regarding the underlying biological mechanisms. Unlike siRNA or shRNA, small molecules directly inhibit or activate target protein, independent from expression level and turnover rate of target protein. Moreover, it is noteworthy that tool compounds collected from a biologically annotated library are optimized to enhance not only the potency against target protein but also target selectivity. Therefore, we hypothesized that MOA analysis of anticancer drugs by using biologically annotated library could become complementary methods for functional genomics.

In this study, we demonstrate that the inhibition of SPT, the rate‐limiting enzyme in sphingolipid synthesis, inhibits the growth of lung cancer cells. We also describe the MOA of SPT inhibitors through a combination study involving a biologically annotated library and SPT inhibitors.

## Materials and methods

### Materials

Reagents were obtained from Life Technologies (Carlsbad, CA, USA) unless otherwise specified. Anti‐COX2 antibody (#12282, WB 1/1000) and anti‐actin antibody (#4970, WB 1/1000) were purchased from Cell Signaling Technologies (Danvers, MA, USA).

### Compounds

Biologically annotated compounds were collected to create a screening compound library. SPT inhibitors were synthesized at Takeda Pharmaceutical Company, Ltd. (Fujisawa, Japan) 23.

### Preparation of human SPT2 enzyme

PCR with specific primers was used to generate cDNA‐encoding human SPT2, and the transcript was subsequently subcloned to generate expression vectors. For preparation of the SPT2 enzyme, FreeStyle293 cells were transfected with human SPT2 expression plasmids and cultured for 3 days. Cells were then homogenized in 50 mm HEPES buffer (pH 7.5) containing 250 mm sucrose, 5 mm EDTA, 5 mm DTT, and Complete, EDTA‐free (Roche Applied Science, Penzberg, Upper Bavaria, Germany). Cell homogenates were centrifuged, and supernatants were harvested. Total membrane fractions were isolated by ultracentrifugation. Pellets were resuspended in 50 mm HEPES buffer (pH 7.5) containing 5 mm EDTA, 5 mm DTT, and Complete, EDTA‐free. Cell lysates were stored at −80 °C. The protein concentration was determined with using the CBB Protein Assay.

### Enzyme assay

Enzyme reactions were run in 20 μL volumes with assay buffer comprising 100 mm HEPES (pH 8.0), 2.5 mm EDTA, 5 mm DTT, and 0.01% bovine serum albumin (fatty acid‐free), and conducted in a 384‐well assay plate. Briefly, 5 μL of a tested compound and 10 μL of 100 μg·mL^−1^ SPT2‐expressed membrane dissolved in assay buffer were mixed and incubated for 60 min. Subsequently, 5 μL of a substrate solution containing 2 mm ‐serine and 20 μm palmitoyl‐CoA in assay buffer was added to start the enzyme reaction. After a 15‐min incubation period at room temperature, the reaction was terminated by adding 20 μL of 2% formic acid. Finally, 40 μL of acetonitrile containing 600 nm C17‐sphinganine was added as an internal standard. High‐throughput online solid‐phase extraction was performed using a RapidFire^®^ 300 device (Agilent Technologies, Santa Clara, CA, USA). Mass spectrometric analysis was performed using an API‐4000™ triple quadrupole mass spectrometer (AB SCIEX, Framingham, MA, USA) in positive SRM mode. The SRM transitions for 3‐ketodihydrosphingosine (reaction product) and C17‐sphinganine were set to 300.5/270.3 and 288.4/60.2, respectively. Analytical data were acquired using analyst software, version 1.5.0 (AB SCIEX), and 300.5/270.3 was divided by 288.4/60.2 for calibration. The IC_50_ values for test compounds were calculated using xlfit software (IDBS, London, UK).

### Cell line

HCC4006 cells were purchased from ATCC (Manassas, VA, USA) and maintained in RPMI supplemented with 10% FBS (Corning Corp., Midland, MI, USA).

### Growth inhibition assay

HCC4006 cells were dispensed into a 384‐well culture plate at a density of 250 cells per well in 40 μL of culture medium and cultured overnight. Subsequently, the cells were treated with 10 μL of a tested compound and cultured for 5 days. The medium was then removed and replaced with 30 μL of CellTiter Glo Luminescent Cell Viability Assay reagent (Promega, Fitchburg, WI, USA). Luminescence was measured on an EnVision device (PerkinElmer, Waltham, MA, USA). The IC_50_ values for test compounds were calculated using graphpad prism 5.0 (GraphPad Software, San Diego, CA, USA).

### Lactate dehydrogenase release

HCC4006 cells were seeded in black 384‐well plates and treated with compounds for 96 h. From each well, 20 μL of cell culture medium was transferred to a 384‐well clear‐bottomed plate (#3680, Corning Corp.); CytoTox 96 Non‐Radioactive Cytotoxicity Assay (Promega Corp.) reagent was added to each well, followed by a 30‐min incubation period; cell variability was measured on a Spectramax Paradigm multiplate reader (Molecular Device Corp., Sunnyvale, CA, USA).

### Combination screening

HCC4006 cells were seeded in black 384‐well plates and pretreated with Biologically annotated compounds for 1 h, after which SPT inhibitors were added for 120 h. CellTiter Glo was added to each well, and cell variability was determined by measuring the firefly luciferase intensity on an EnVision device.

### Western blotting

Cells were collected and lysed in RIPA buffer (Wako Pure Chemicals, Osaka, Japan) containing protease inhibitor cocktail (Roche, Basel, Switzerland). Lysates was boiled with SDS sample buffer (Bio‐Rad, Hercules, CA, USA) containing 100 μm dithiothreitol. Samples were electrophoresed on 5–20% SDS polyacrylamide gels (ATTO, Tokyo, Japan), transferred to polyvinylidene fluoride membranes using an iBLOT apparatus (Thermo Fisher Scientific, Waltham, MA, USA), and immunostained using the indicated antibodies.

### Pathway enrichment analysis

We used the Ingenuity Pathway Analysis (IPA) system for canonical pathway enrichment analysis to perform functional enrichment tests of target candidate genes linked to the hit compounds.

### Statistical analysis

Values are presented as means ± SD. Statistical significance among groups was determined using an ANOVA followed by Dunnett's test. A *P* value <0.05 was considered statistically significant.

Caspase 3/7 assay, ROS Glo assay, siRNA transfection, and Monoacylglycerol lipase assay are available as Supporting Information, Appendix S1.

## Results and Discussion

### SPT inhibitors attenuate lung cancer cell growth

**Figure 1:**
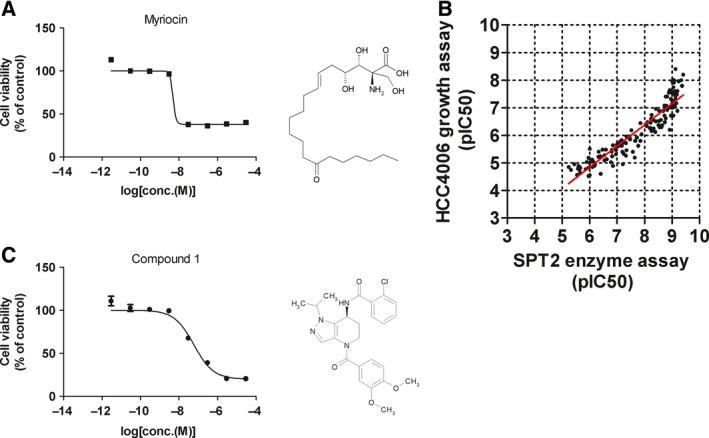
Chemical structure and growth inhibitory activities of serine palmitoyl transferase (SPT) inhibitors. (A) HCC4006 cells were treated with myriocin for 120 h. Cell viability was measured using CellTiter Glo. The chemical structure of myriocin is also described. Values are reported as means ± SEM in arbitrary units (*n* = 4). (B) HCC4006 cells were treated with Compound 1 for 120 h. Cell viability was measured by CellTiter Glo. The chemical structure of Compound 1 is also described. Values are reported as means ± SEM in arbitrary units (*n* = 4). (C) Relationship of HCC4006 cell growth inhibition with SPT inhibitory activity. HCC4006 cells were treated with a range of doses of SPT inhibitor for 120 h. The pIC_50_ values, indicating growth inhibitory activity, of each compound are plotted against the *in vitro* SPT2 enzyme inhibitory activity.

Previous studies suggested that SPT inhibition suppressed the growth of both melanoma and lung cancer cells 24, 25. We found that the lung cancer cell line HCC4006 was sensitive to myriocin, a known SPT inhibitor (Fig. 1A). Therefore, we synthesized 137 pyrazolopyridine derivatives as SPT inhibitors and used these to validate the relationship between *in vitro* SPT activity inhibition and cancer cell growth. We confirmed that the inhibition of HCC4006 cancer cell growth correlated well (*R* ^2^ = 0.87) with the *in vitro* inhibition of SPT2 enzyme activity, suggesting that SPT inhibition is responsible for HCC4006 cancer cell growth inhibition (Fig. 1B). In this study, we utilized compound 1 as a chemical probe against SPT. Compound 1 inhibited SPT2 with an IC_50_ value of 0.8 nm in an *in vitro* enzyme assay and suppressed HCC4006 cell growth with an IC_50_ value of 59 nm (Fig. 1C).

### SPT inhibitor induces necrosis‐dependent cell death in HCC4006 cells

**Figure 2:**
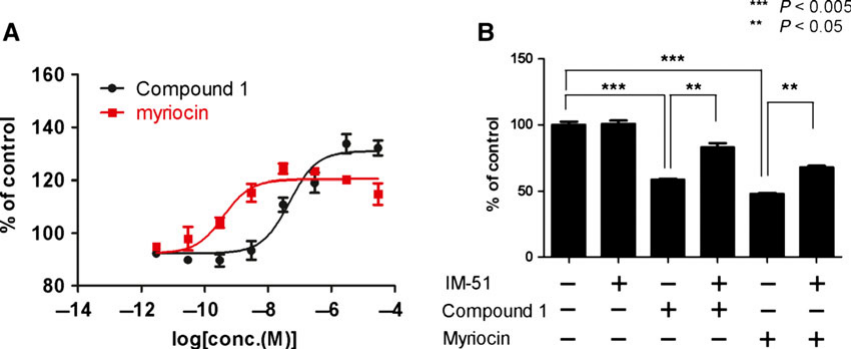
Characterization of the cell death mechanism of action. (A) HCC4006 cells were treated with various doses of Compound 1 for 96 h. Necrosis activity was measured by a lactate dehydrogenase (LDH) release assay. Values are reported as means ± SEM in arbitrary units (*n* = 4). (B) HCC4006 cells were cotreated with 1 μm Compound 1 or 300 nm myriocin and 10 μm necrosis inhibitor IM‐51 for 120 h. Cell viability was measured by CellTiter Glo. Values are reported as means ± SEM in arbitrary units (*n* = 4).

Although SPT inhibition was shown to induce growth inhibition in HCC4006 cancer cells, the underlying MOA remained unclear. Cell death can be largely classified as follows, according to morphological and biochemical characteristics: apoptosis or programmed cell death; nonapoptotic cell death such as necrosis; and ferroptosis, an increasingly recognized and well‐regulated cell death mechanism 26, 27. To understand the MOA, we examined which types of cell death were induced by SPT inhibitors using a well‐characterized assay system and specific inhibitors against each pathway. First, effects against the apoptotic pathway were examined, as previous reports suggested that SPT inhibition induced apoptotic signals 25. However, under our assay conditions, we confirmed that caspase 3/7 cleavage was activated by compound 1 only at concentrations exceeding 3 μm, but was not activated with myriocin treatment, suggesting that the observed caspase 3/7 activity at high concentrations of compound 1 might represent an off‐target effect (Fig. S1A, Supporting Information). We also confirmed that treatment with the pan‐caspase inhibitor z‐vad did not attenuate SPT inhibitor‐induced growth inhibition (Fig. S1B). Taken together, these observations suggest that apoptosis is not involved in SPT inhibitor‐induced cell growth inhibition. Second, we evaluated whether SPT inhibitor treatment would induce necrosis. Necrosis is an apoptosis‐independent cell death mechanism characterized by a disruption of the cell membrane structure and subsequent release of cellular components to the extracellular medium. Treatment with compound 1 and myriocin induced lactate dehydrogenase (LDH) release in a dose‐dependent manner with respective EC_50_ values of 47 nm and 0.4 nm (Fig. 2A), indicating good agreement with the IC_50_ values for cell growth (59 nm and 4 nm, respectively) and suggesting that SPT inhibition leads to necrosis. We further confirmed that the known necrosis inhibitor IM‐54, which was originally identified as a suppressor of hydrogen peroxide‐induced necrosis 28, attenuated SPT inhibitor‐induced cell death (Fig. 2B). Third, we examined whether SPT inhibitor treatment would induce ferroptosis. Ferroptosis is a newly identified type of cell death involving the iron‐dependent accumulation of reactive lipid species 29. We confirmed that treatment with SPT inhibitors induced the generation of reactive oxygen species (ROS), a hallmark of ferroptosis, whereas treatment with ferrostatin‐1, a well‐characterized ferroptosis inhibitor, did not attenuate Compound 1‐induced cell growth inhibition (Fig. S1C,D). These data suggest that ROS generation is a secondary effect of SPT inhibitor treatment and that SPT inhibitor‐induced cell growth inhibition is independent of ferroptosis. These results collectively indicate that SPT inhibitors suppress cell growth via the necrotic pathway.

### Compound combination screening using a biologically annotated library with SPT inhibitors

**Figure 3:**
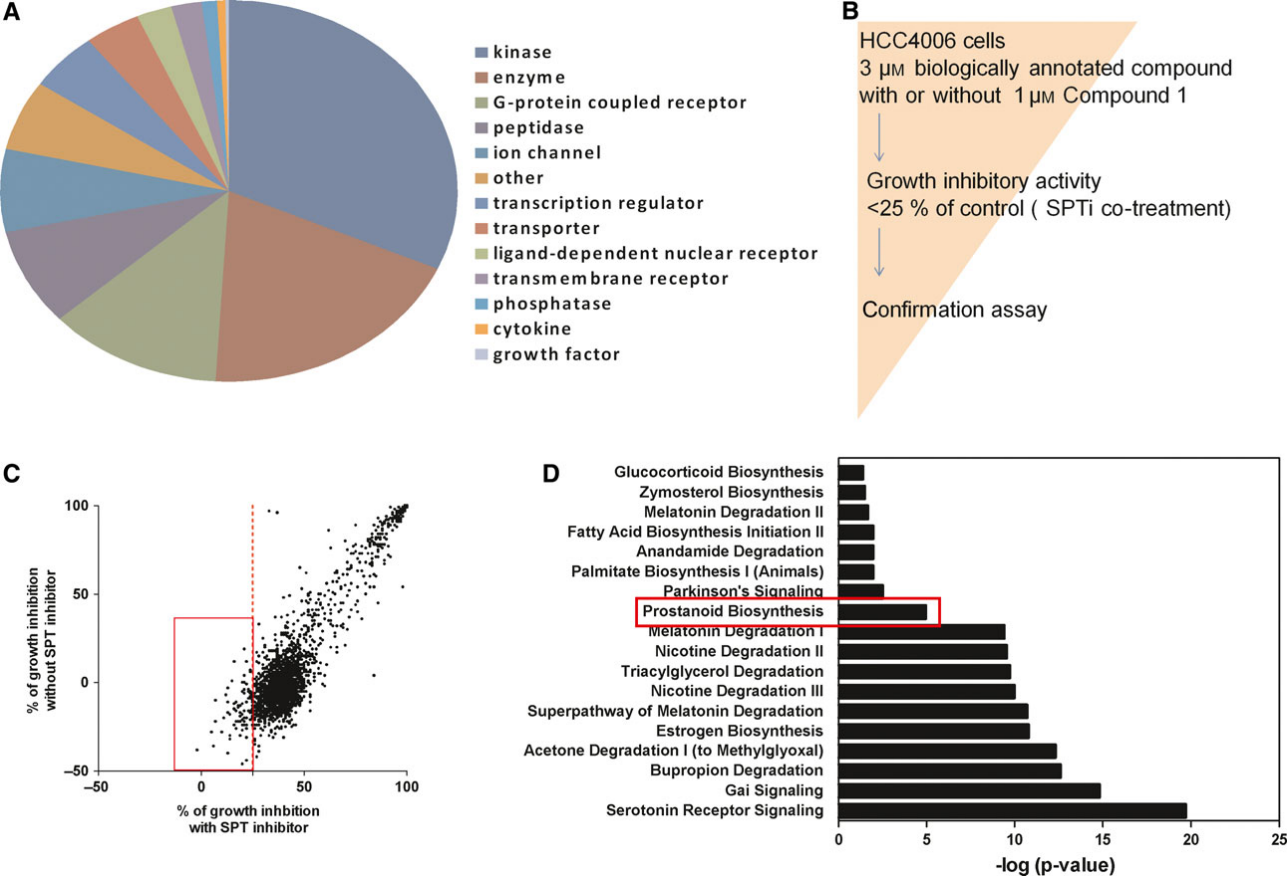
Unbiased screening with a biologically annotated library. (A) Composition of library used for combination screening. (B) Scheme of combination library screening. (C) HCC4006 cells were treated with the biologically annotated library components in the presence or absence of 1 μm Compound 1 for 120 h. The inhibitory activity of each compound is plotted. (D) Results of an IPA software‐based pathway enrichment analysis.

A recent study illustrated that functional genomics studies involving siRNA or shRNA could be a useful approach to the elucidation of unknown MOA of targeted compounds 30. However, suppression of a single gene might be overwhelmed by the compensatory activity of structurally related subtypes 16 and, for siRNA studies in particular, the efficiency of knockdown varied according to the target protein and, in most cases, partial knockdown did not affect the desired phenotype; in addition, off‐target effects of siRNA are frequently observed 14, 15. To overcome these obstacles, we performed an unbiased combination study using a biologically annotated library with SPT inhibitors. The concept of a biologically annotated library has been proposed by several pharmaceutical companies 17, 18, 19, 20. We collected approximately 3000 compounds to form our biologically annotated library. Our criteria for the selection of compounds were *in vitro* pharmacological activity with IC_50_ or EC_50_ value of less than or equal to 1 μm on each target protein, which is based on the results of cell‐free and cell‐based assays with multiple types such as functional and binding assays, as shown in Fig. 3A and Table S1. Consequently, our biologically annotated compound library targets approximately 1500 unique proteins, each of which is often annotated by multiple compounds to avoid the misinterpretation of the results caused by off‐target effects of small molecules. In fact, 70% of target protein information is annotated by two or more compounds. The remaining 30% covered by a single compound for each is still included, since functional genomics approaches (e.g., CRISPR, shRNA) would strengthen the hypothesis derived from the small molecule‐based approaches. In our combination study, the SPT inhibitor concentration was set to 1 μm, which was expected to exhibit maximal growth inhibitory activity when used with biologically annotated library compounds at a concentration of 3 μm; the latter was expected to fully regulate the target protein activity. We screened biologically annotated library at 3 μm concentration with or without 1 μm SPT inhibitor (Fig. 3B). We identified 33 hit compounds that mitigated SPT inhibitor‐induced cell death (Fig. 3C and Table 1).

**Table 1:** List of hit compounds identified via combination screening

Compound name	Mechanism of action	Target class
Orlistat	Arachidonic acid production	Enzyme
CHEMBL130098	Hormone‐sensitive lipase	Enzyme
CHEMBL1082517	Lysosomal acid lipase (LIPA)	Enzyme
JZL184	Monoacyl glycerol lipase(MAGL)	Enzyme
Flumetasone	COX2	Enzyme
Beclomethasone	COX2	Enzyme
Celecoxib	COX2	Enzyme
Rutaecarpine	COX2	Enzyme
Econazole	Ergosterol synthesis	Enzyme
CHEMBL557129	CDC25B	Enzyme
CHEMBL1471965	PSMD14	Enzyme
Necrostatin‐1	RIPK1	Kinase
CHEMBL1462325	AHR	Ligand‐dependent nuclear receptor
CHEMBL334330	RARB	Ligand‐dependent nuclear receptor
PRIMA‐1	p53	Transcription regulator
pubchem2115839	STAT3	Transcription regulator
CID:|4283428	KLF5	Transcription regulator
CHEMBL165418	ABCC1	Transporter
Bromocryptine	D2R	GPCR
MK‐329	CCKAR	GPCR
Loxapine	5HTR	GPCR
N‐methylquipazine	5HT3A	GPCR
CP‐135807	5‐HT1D	GPCR
CHEMBL133534	MT1	GPCR
Promethazine	H1R	GPCR
Tripelennamine	H1R	GPCR
Tolterodine	M2/M3	GPCR
Montelukast	Leukotriene receptor	Transmembrane receptor
4EGI‐1	eIF4E/eIF4G interaction inhibitor	Other
Pentamidine	S100PRAGE	Other
Torcetrapib	Cholesterol ester transfer protein	Other
Miltefosine	Phospholipid antimicrobial drug	Other
Azaguanine‐8	Guanine analog	Other

### Upregulation of COX‐2 expression triggers necrosis in SPT inhibitor‐treated cells

**Figure 4:**
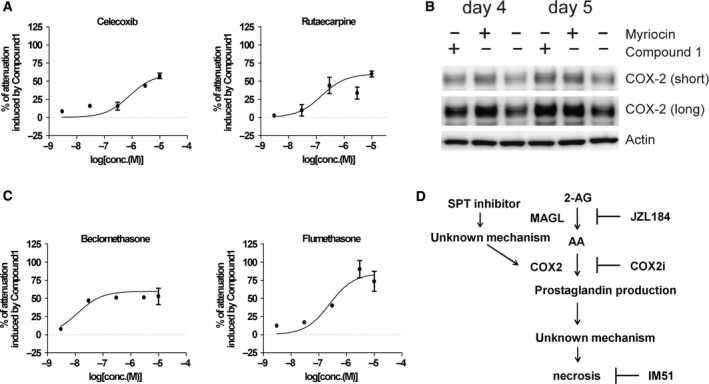
Validation of the combination library screening results. (A) HCC4006 cells were treated with various concentrations of the COX‐2 selective inhibitors celecoxib and rutaecapine together with 1 μm Compound 1 for 120 h. Cellular viability was measured with CellTiter Glo. Values are reported as means ± SEM in arbitrary units (*n* = 4). (B) HCC4006 cells were treated with 1 μm Compound 1 or 300 nm myriocin for the indicated times. Cell lysates (5 μg) were separated via 4–20% polyacrylamide gel electrophoresis, and COX‐2 and beta‐actin protein levels were detected via immunoblotting. (C) Cells were treated with various concentrations of COX‐2 expression inhibitors and 1 μm Compound 1 for 120 h. Cellular viability was measured using CellTiter Glo. Values are reported as means ± SEM in arbitrary units (*n* = 4). (D) Summary of the molecular mechanism of action of SPT inhibitors.

Pathway enrichment analysis, using IPA pathway enrichment software, was performed to reveal essential pathways related to SPT inhibitor‐induced cell death, and 18 pathways were nominated as candidate pathways (Fig. 3D). We focused on the prostanoid biogenesis pathway because we noticed that 4 of the 33 hit compounds were related to COX‐2, which catalyzes the conversion of arachidonic acid to prostanoid. Two selective COX‐2 inhibitors, celecoxib and rutaecapine, are included in this category 31, and were found to dose‐dependently attenuate Compound 1‐mediated growth inhibition (Fig. 4A). COX‐2 is an inducible family protein that is expressed at low levels under basal conditions; expression of this protein can be induced by a particular stimuli, leading to the generation of prostaglandin products 32, 33. We examined whether treatment with SPT inhibitors would induce COX‐2 expression, thus validating our combination library‐screening findings, and confirmed that treatment with SPT inhibitors induced COX‐2 expression after 96 h (Fig. 4B). We also confirmed that beclomethasone and flumethasone suppressed SPT inhibitor‐mediated cell death (Fig. 4C). These two compounds were previously reported as suppressors of COX‐2 expression 34. These results strongly suggest that Compound 1‐induced cell growth inhibition is mediated by COX‐2 function. Next, we reanalyzed the results of our biologically annotated library screening and found that JZL184 suppresses Compound 1‐induced cell growth inhibition. JZL184 is an irreversible inhibitor for monoacylglycerol lipase (MAGL), the primary enzyme responsible for degrading the endocannabinoid 2‐arachidonoylglycerol (2‐AG) to arachidonic acid 31. We measured the inhibitory activities of two other lipase inhibitors, CHEMBL130098 and CEHMBL1082517 against MAGL because arachidonic acid metabolism is likely to be the key pathway for Compound 1‐induced cell growth inhibition. As expected, both compounds inhibited *in vitro* MAGL enzyme activity (Table S2). These results indicate that seven compounds out of 33 hit compounds identified via combination library screening were related to arachidonic acid metabolism. These observations strongly support the validity of our biologically annotated library‐screening strategy. Finally, we performed a knockdown experiment using COX‐2 or MAGL siRNA to exclude the possibility of off‐target effect of annotated compounds. Treatment of COX‐2 or MAGL siRNA suppressed Compound 1‐induced cell growth inhibition (Fig. S2), and these results also support the importance of COX‐2‐ and MAGL‐related pathway for SPT inhibitor‐mediated cancer cell death.

In summary, we conducted a combination screening of compounds using a biologically annotated library to reveal the MOA of SPT inhibition. Accordingly, we found that COX‐2 expression was upregulated by SPT inhibition. Although the mechanism by which COX‐2 expression is induced remains unclear, COX‐2 induction was critical for SPT inhibition‐induced cell death (Fig. 4D). Our results present the possibility that the expression level of MAGL or COX2 in lung cancer patients could be one of the candidates of the patient stratification marker. A more detailed analysis will be the subject of further study. Finally, we emphasize that our combination approach involving a biologically annotated library could be widely applicable to the investigation and discovery of the MOA of other types of anticancer drugs. Our compound combination screening using a biologically annotated library for anticancer drug MOA analysis could provide novel findings on target‐related pathways and be applicable as a complementary method for functional genomics‐based MOA analysis.

## Author contributions

OS and RA conducted all experiments. KK analyzed data. OK synthesized SPT inhibitors. OS wrote the manuscript. TK and HI conceived the study and participated in its design and in drafting of the manuscript. All authors have read and approved the final manuscript.

## Supporting information


**Fig. S1.** (A) HCC4006 cells were treated with various concentrations of Compound 1 or myriocin for 96 h. Caspase 3/7 activity was measured using a Caspase 3/7 Glo assay. (B) HCC4006 cells were treated with various concentrations of Compound 1 or myriocin and 20 µm z‐VAD for 120 h. Cellular viability was measured using CellTiter Glo. (C) HCC4006 cells were treated with various concentrations of Compound 1 or myriocin for 96 h. Intracellular reactive oxygen species (ROS) production was measured using a ROS Glo assay. (D) HCC4006 cells were treated with various concentrations of Compound 1 or myriocin with 10 µm Ferrostatin‐1. Cellular viability was measured by CellTiter Glo.
**Fig. S2.** (A) HCC4006 cells were cotreated with 6 nm COX‐2, MAGL, or control siRNA with 3 µm Compound 1 for 72 h. Cellular viability was measured using CellTiter Glo. (B) HCC4006 cells were treated with 6 nm COX‐2, MAGL, or control siRNA for 48 h. Cells lysates were subjected to measure expression level of COX‐2 and MAGL by qPCR. Relative knockdown efficiency was calculated by delta–delta CT method.
**Table S1.** Composition of library used for combination screening.
**Table S2.** Summary of inhibitory activity against monoacylglycerol lipase (MAGL).
**Appendix S1.** Materials and methods.
