# Supplementary material for: Using a biologically annotated library to analyze the anticancer mechanism of serine palmitoyl transferase (SPT) inhibitors
Source: FEBS Open Bio. 2017 Feb 13;7(4):495–503. doi: 10.1002/2211-5463.12196 (PMC5377399; doi:10.1002/2211-5463.12196)
Supplement: Supplementary file 1 — Fig. S1. (A) HCC4006 cells were treated with various concentrations of Compound 1 or myriocin for 96 h. Caspase 3/7 activity was measured using a Caspase 3/7 Glo assay. (B) HCC4006 cells were treated with various concentrations of Compound 1 or myriocin and 20 µm z‐VAD for 120 h. Cellular viability was measured using CellTiter Glo. (C) HCC4006 cells were treated with various concentrations of Compound 1 or myriocin for 96 h. Intracellular reactive oxygen species (ROS) production was measured using a ROS Glo assay. (D) HCC4006 cells were treated with various concentrations of Compound 1 or myriocin with 10 µm Ferrostatin‐1. Cellular viability was measured by CellTiter Glo. Fig. S2. (A) HCC4006 cells were cotreated with 6 nm COX‐2, MAGL, or control siRNA with 3 µm Compound 1 for 72 h. Cellular viability was measured using CellTiter Glo. (B) HCC4006 cells were treated with 6 nm COX‐2, MAGL, or control siRNA for 48 h. Cells lysates were subjected to measure expression level of COX‐2 and MAGL by qPCR. Relative knockdown efficiency was calculated by delta–delta CT method. Table S1. Composition of library used for combination screening. Table S2. Summary of inhibitory activity against monoacylglycerol lipase (MAGL). Appendix S1. Materials and methods. [file FEB4-7-495-s001.docx]

**Supplementary Figures and Tables**

Supplementary Figure 1.

 (A) HCC4006 cells were treated with various concentrations of Compound 1 or myriocin for 96 hours. Caspase 3/7 activity was measured using a Caspase 3/7 Glo assay.

(B) HCC4006 cells were treated with various concentrations of Compound 1 or myriocin and 20 µM z-VAD for 120 hours. Cellular viability was measured using CellTiter Glo.

(C) HCC4006 cells were treated with various concentrations of Compound 1 or myriocin for 96 hours. Intracellular reactive oxygen species (ROS) production was measured using a ROS Glo assay.

(D) HCC4006 cells were treated with various concentrations of Compound 1 or myriocin with 10 µM Ferrostatin-1. Cellular viability was measured by CellTiter Glo.

Supplementary Figure 2.

 (A) HCC4006 cells were co-treated 6 nM COX-2, MAGL, or control siRNA with 3 µM Compound 1 for 72 hours. Cellular viability was measured using CellTiter Glo.

(B) HCC4006 cells were treated with 6 nM COX-2, MAGL or control siRNA for 48 hours. Cells lysates were subjected to measure expression level of COX-2 and MAGL by qPCR. Relative knock-down efficiency was calculated by delta-delata CT method.

Supplementary Table 1.

Composition of library used for combination screening.

| Type(s) | Unique Target |
| --- | --- |
| kinase | 424 |
| enzyme | 318 |
| G-protein coupled receptor | 180 |
| ion channel | 143 |
| peptidase | 112 |
| other | 90 |
| transporter | 77 |
| transcription regulator | 68 |
| transmembrane receptor | 37 |
| ligand-dependent nuclear receptor | 34 |
| phosphatase | 18 |
| cytokine | 9 |
| growth factor | 5 |
| translation regulator | 1 |
| Total | 1516 |

Supplementary Table 2.

Summary of inhibitory activity against monoacylglycerol lipase (MAGL).

| **Compound Name** | **Mechanism of Action** | **MAGL inhibitory activity (IC50)** |
| --- | --- | --- |
| CHEMBL130098 | Hormone sensitive lipase | 14 nM |
| CHEMBL1082517 | Lysosomal acid lipase (LIPA) | 3100 nM |
| JZL184 | Monoacyl glycerol lipase(MAGL) | 10 nM |

**Supplementary Materials and Methods**

Reagents

Z-vad, caspase 3/7 Glo reagent, and ROS Glo reagent were purchased from Promega Corp (Fitchburg, WI, USA). Ferrostatin-1 was purchased from SIGMA (St. Louis, MO, USA).

Caspase 3/7 assay

HCC4006 cells were seeded in white 384-well plates (#3570, Corning Corp., Corning, NY, USA) and treated with the indicated compounds for 96 hours. Caspase 3/7 Glo (Promega Corp.) was added to each well, and cell viability was determined by measuring the firefly luciferase intensity on an EnVision device (PerkinElmer, Waltham, MA, USA).

ROS Glo assay

HCC4006 cells were seeded in white 384-well plates (#3570, Corning Corp.) and treated with the indicated compounds for 96 hours. ROS-Glo (Promega Corp.) substrate was added to each well, after which cells were incubated at 37ºC for three hours. Detection solution was then added to each well, and ROS generation was determined by measuring the firefly luciferase intensity on an EnVision device.

siRNA transfection

HCC4006 cells were transfected with a final concentration of 6 nM siRNA. RNAiMAX was used for the reverse transfection according to the manufacturer’s instructions. To silence COX-2 or MAGL, specific siRNAs (Silencer® Select Pre-designed siRNA from Life Technologies; s11472, s11473, and s11474 for COX-2, s22379, s22380, and s22381 for MAGL, and negative control siRNA #1 for control) were used. Forty-eight hours after siRNA transfection, cells were harvested for real-time RT-PCR analysis using 1-step cells to CT reagent, and the knockdown of each mRNA was confirmed using TaqMan probes (Hs00228159_m1 for COX-2, Hs00996004_m1 for MAGL, and Hs01060665_g1 for Actin as an internal control).

Monoacylglycerol lipase (MAGL) assay

Compounds were dissolved in DMSO and subsequently diluted in enzyme reaction buffer (10 mM Tris-HCl, pH 7.5, 1 mM EGTA, 0.025% (w/v) Triton X-100, 0.01% bovine serum albumin [BSA]). Recombinant human MAGL was diluted in enzyme reaction buffer to a concentration of 7.5 ng/mL. Five microliters of compound solution were added to each well of a 384-well assay plate, and 5 µL of enzyme mixture were added per well. The mixtures were incubated at room temperature for 60 min. Next, 5 µL of substrate solution (150 µM 2-arachidonylglycerol) was added to each well, and the mixture was incubated at room temperature for 10 min. The reaction was stopped by adding 10 µL of 2% formic acid and 50 µL of acetonitrile containing 3 µM arachidonic acid-d8 (Cayman Chemical). Arachidonic acid production was detected via RapidFire mass spectrometry and corrected to arachidonic acid-d8.
